# Isolated Chest Wall Necrotizing Fasciitis: An Unusual Fatal Manifestation of Extrapulmonary Tuberculosis

**DOI:** 10.7759/cureus.20585

**Published:** 2021-12-21

**Authors:** Satya P Meena, Netrananda Acharya, Prakash C Kala, Mahaveer Rohda

**Affiliations:** 1 General Surgery, All India Institute of Medical Sciences, Jodhpur, Jodhpur, IND; 2 Plastic Surgery, All India Institute of Medical Sciences, Jodhpur, Jodhpur, IND

**Keywords:** chest, extrapulmonary tuberculosis, necrotizing fasciitis, osteomyelitis, infection

## Abstract

Primary tuberculosis of the chest wall is a rare disease and very difficult to diagnose without clinical suspicion. Here, we present an unusual case of necrotizing fasciitis due to an aggressive form of chest wall tuberculosis. A 22-year-old male presented in emergency with acute-onset swelling and redness over the right side of the neck and chest wall. He had no history of any drug reaction, trauma, and unknown bite. The patient underwent aggressive debridement followed by split-thickness graft under intensive care monitoring. Radiological imaging and Ziehl-Neelsen (ZN) staining of pleural fluid revealed no evidence of pulmonary tuberculosis. Special investigations such as cartridge-based nucleic acid amplification test and ZN staining from pathological skin or subcutaneous tissue revealed active tuberculosis; therefore, anti-tubercular drugs were started.

## Introduction

Cervical lymphadenopathy as extrapulmonary tuberculosis (EPTB) accounts for every sixth patient with tuberculosis in India. However, bone and joint involvement are seen in 1-3% of tubercular patients [1]. Primary tuberculosis of the chest wall is an uncommon chronic disease and is usually difficult to manage [2]. Necrotizing fasciitis of the neck and face is an unusual complication of the dental or subcutaneous disease and has a fulminant course in immunocompromised patients [3]. An acute-onset aggressive form of EPTB is a very rare fatal presentation. Preoperative ultra-short chemotherapy along with surgical debridement effectively shortens hospitalization and increases patient compliance for EPTB [4].

## Case presentation

**Figure 1:**
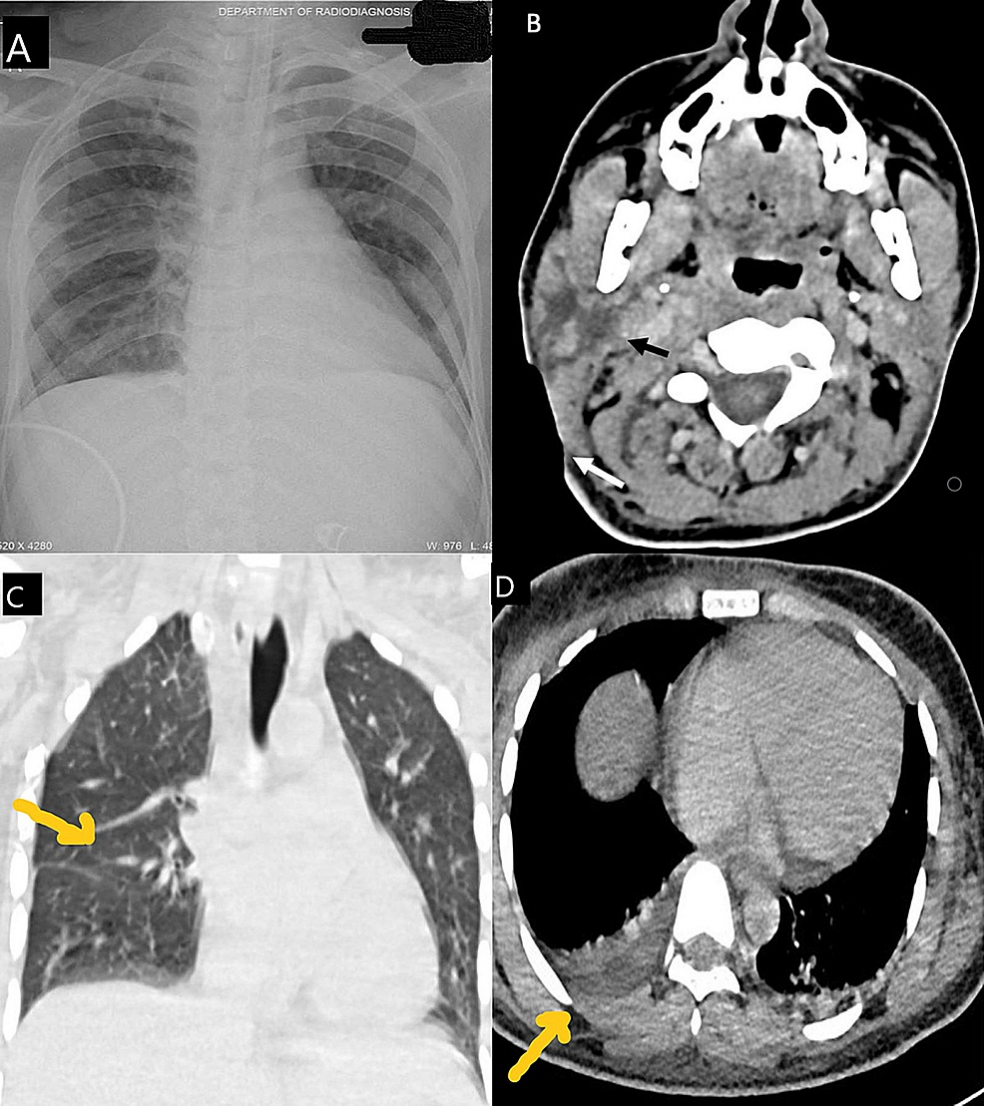
Radiology findings. A: X-ray of the chest: grossly normal. B: CECT of the neck (axial): an arrow showing the irregular, well-defined, non-enhancing area into the neck spaces extending to the parotid area, and another arrow showing the soft-tissue defect in the neck region. C: CECT of the chest (coronal): lung window image revealing normal bilateral lung parenchyma. D: CECT of the chest (axial): mediastinal window image showing a mild right-sided pleural effusion. CECT: contrast-enhanced computed tomography

A 22-year-old male presented in the emergency department with acute onset of swelling and redness over the right side of the neck and chest wall for the last three days. He had features of septicemia such as drowsiness or Glasgow Coma Scale score of 11/15, respiratory rate of 26 breaths per minute, pulse rate of 130 beats per minute, blood pressure of 84/56 mmHg, and urine output of 15 mL/hour. He had no history of chronic disease, drug reaction, trauma, unknown bite, or significant familial disease. Blood investigations revealed low hemoglobin of 7.6 g/dL, raise leukocyte count of 28000/mm^3^, low albumin of 2.2 g/dL, raised serum creatinine of 2.23 mg/dL, serum urea of 174 mg/dL, and low sodium of 125 mEq/L. Serological markers including erythrocyte sedimentation rate (95 mm/hour) and procalcitonin (25.2 ng/mL) were higher. X-ray of the chest was grossly normal (Figure 1A), and contrast-enhanced computed tomography (CECT) of the neck revealed irregular, well-defined, hypodense, non-enhancing area in the right parotid gland with extension into the neck spaces, larynx, and subcutaneous planes (Figure 1B). CECT of the chest revealed mild effusion in bilateral pleural space secondary to acute infection and no evidence of lymphadenopathy or osteomyelitis (Figures 1C, 1D).

**Figure 2:**
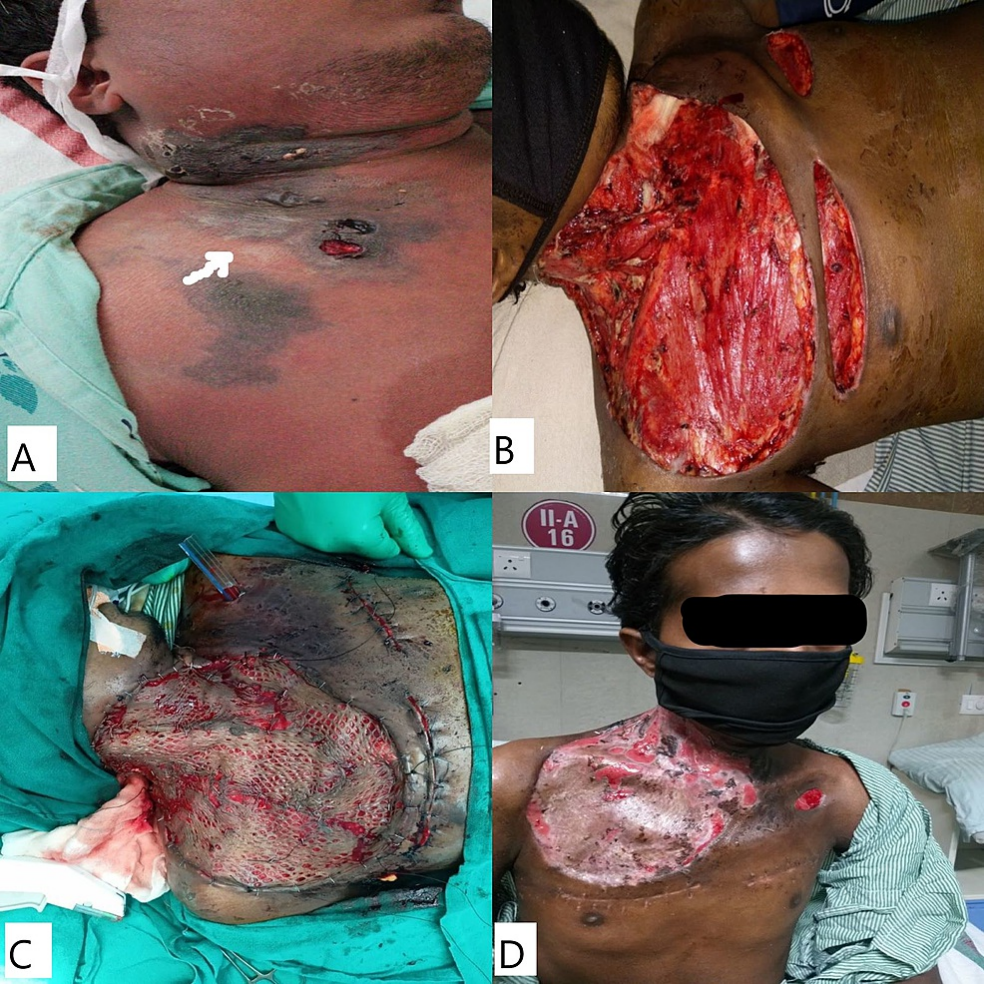
Clinical image. A: clinical image showing necrotizing skin lesion over the neck and chest wall. B: post-debridement image. C: split-thickness skin graft application. D: follow-up 15 days after discharge.

Ziehl-Neelsen (ZN) staining from pleural fluid was negative for acid-fast bacillus (AFB bacilli). The patient was managed in the intensive care unit with ventilator support due to acute respiratory distress syndrome. He was diagnosed with acute progressive necrotizing fasciitis with multiple organ dysfunction syndromes due to an unknown cause of septicemia. He underwent multiple aggressive debridements of the neck and chest wall (Figure 2).

The cartridge-based nucleic acid amplification test (CBNAAT) and ZN staining revealed positive mycobacteria and AFB bacilli for pathological tissue. Therefore, the pus collected in the parotid region and neck space was likely secondary to tubercular infection. A split-thickness graft was applied in multiple fractions after three weeks of anti-tubercular chemotherapy. The patient had a long hospital stay due to the difficult location and complexity of the ulcer over the neck. He was followed up for six months in good clinical condition after discharge.

## Discussion

Necrotizing fasciitis is a rare outcome of EPTB [4]. Chest wall abscess following EPTB usually occurs as a solitary lesion, mostly at the margins of the sternum and in the shafts of ribs [5]. However, in this case, subcutaneous and superficial fascial layers of the neck and chest wall were involved without evidence of osteomyelitis. Tuberculosis is usually a chronic lesion with a more indolent and latent period; however, our patient had an aggressive progression. Usually, a radiological or clinical sign of underlying pulmonary tuberculosis is present with or without bony involvement; however, in our patient, there was absolutely no evidence of pulmonary tuberculosis or osteomyelitis on imaging [6]. A high index of suspicion is needed if pulmonary tuberculosis is not present. Necrotizing fasciitis infection associated with the face, cervical, orbital wall, chest wall involvement has higher morbidity and mortality, thus requiring aggressive surgical management with anti-tubercular drugs [7]. The patients may require intensive care monitoring with a tracheostomy. Sometimes thoracoscopic drainage or thoracotomy also plays a role in preventing sepsis [8].

Initially, the diagnosis is made as acute necrotizing fasciitis due to an unknown bacterial or fungal infection, likely caused by mycobacteria as it is endemic in India. Therefore, cultures for different pathogens, soft tissue biopsy, as well as specific nucleic tests are recommended to identify a definitive cause. In our patient, although radiological imaging did not indicate tuberculosis, we were able to make the definitive diagnosis after specific investigations.

According to previous literature, one case of primary tuberculous dermatomyositis over the left thigh has been reported as a chronic manifestation [6]. In addition, three cases of necrotizing fasciitis over the gluteal and upper limb have been reported as a complication of miliary pulmonary tuberculosis [3,9]. Another case study showed chest wall necrotizing fasciitis following intercostal tube removal in a known case of pulmonary tuberculosis [10]. To date, no single case has been reported as primary or extrapulmonary tubercular necrotizing fasciitis over the neck or chest wall.

## Conclusions

Primary or isolated tubercular necrotizing fasciitis should be considered as a differential diagnosis for acute soft tissue infection over the neck and anterior chest wall, especially in endemic countries like India. Feature of osteomyelitis may be absent in the early phase of EPTB. Aggressive debridement along with anti-tubercular drugs are the mainstay of treatment.
